# Engaging in the good with technology: a framework for examining positive technology use

**DOI:** 10.3389/fpsyg.2023.1175740

**Published:** 2023-08-15

**Authors:** Andrew Villamil, Saeideh Heshmati

**Affiliations:** Department of Psychology, Claremont Graduate University, Claremont, CA, United States

**Keywords:** positive psychology, cyberpsychology, positive technology, digital technology, digital media, smartphones, social media, virtual reality

## Abstract

The focus on the negative side of technology has become a prominent factor in the understanding of the interactions between humans and technology. However, there is a positive side to technology use that has been less investigated in scientific research. Well-being researchers have determined that it is not just the absence of negative emotions or experiences, but rather the presence and frequency of positive ones that matter most. Therefore, despite the scarcity of research on the positive side of technology, the present conceptual paper focuses on how technology may be used for the *good* to produce psychological benefits (e.g., greater happiness, lower loneliness, higher peer endorsement). Based on existing literature, we posit at least three directions for good interactions with technology: (1) “seeing good” by focusing on positive visual cues through technology use; (2) “feeling good” by focusing on good feelings that arise from technology use; and (3) “doing good” by focusing on positive actions that can be enacted via technology use. Based on the synthesis of these three components, we propose a framework for technology laden engagement in the good, dubbed as, the *Engagement in the Good with Technology (EGT) Framework*. Through this framework, we explain how these three distinct aspects of seeing, feeling, and doing good can co-occur and be interrelated, and in turn potentially lead to upward spirals of positive outcomes.

## Introduction

Most research into technology has focused on the negative aspects of technology use. Although much research finds detrimental impacts of technology use on people’s psychological well-being, other studies show mixed findings. Less research has been conducted on how technology is used in a positive way that can in turn lead to positive outcomes for the person and their health and well-being. Similar to almost any other tool, there are positive and negative ways one can experience technology that can be beneficial or detrimental to them. Digital technology has experienced rapid adoption across several generational cohorts and the effects of this usage are still not fully understood. In fact, the COVID-19 pandemic alone has shifted perspectives on technology use. While younger adults are the predominant users of technology, research conducted by the AARP has demonstrated that older adults (44%) view technology positively and as a primary means of connection (Kakulla, 2021). Additionally, over 80% of adults 50 and over depend on technology to connect with family and friends through texting, emailing, video chatting, and social media. Overall, a considerable proportion of young adults and older adults’ time is spent online where they are constantly engaging with streams of information, images, sensations, and experiences that may influence their mental health, development, and well-being.

Even though technology use is becoming pervasive and more research studies are focusing on the effects of technology, there still remains a plethora of questions around the benefits that technology use might have. For instance, how can technology be used to elicit positive emotions such as joy, awe, self-transcendence, love, and/or positive values? Even less research has been conducted on understanding the mechanisms of technology that support collaborative behavior between people across different backgrounds and beliefs. How can positive actions be enacted through technology in manners that support resource building, positive discussions, or prosocial behaviors, which in turn strengthen connections and increase positive engagement? While social media presents some positive outcomes as it relates to maintaining relationships, there is a lack of concrete research into the positive uses of technology that can reduce loneliness, depression, anger, substance abuse, radicalization, hate, or anxiety. This gap requires a broader understanding of how positive mechanisms may lead to positive outcomes by engaging in the good with technology.

The complicated nature of technology use has invited scholars from disciplines such as philosophy to better understand how societies can understand the good sides of technology or, as Coeckelbergh refers to “the good society with technology” (2018). In fact, philosophers focusing on technology and its use have explored the nature of technology and determined that humans shape the tools they use. Humans decide how these tools are utilized and in turn, determine if it is used for the good or the bad—this may depend on the community and social influence on the perceptions of values toward technology (Coeckelbergh, 2018). In this dynamic relationship between humans and technology, fostering ethical considerations and promoting critical engagement with technological advancements becomes paramount. By actively recognizing and embracing our role as shapers of technology, we can collectively strive toward harnessing its potential for positive impact and shaping a future where technology serves the greater good.

Based on existing literature, there are at least three directions where good interactions with technology have been individually examined: *Seeing good, Feeling Good,* and *Doing Good*. One line of research has focused on the interactions and effects of positive visual cues through technology [i.e., seeing good (Janicke-Bowles et al., 2018); moral elevation (Haidt, 2000); memes (Myrick et al., 2022)]. Another line of research has focused on good feelings as a result of interacting with technology [i.e., “feeling good” via increases in positive affect (Diener, 1984); broaden and build theory (Fredrickson and Joiner, 2002); prosocial media (Greitemeyer, 2009); social media (Sherman et al., 2016)]. Lastly, a line of research focuses on positive actions through technology [i.e., “doing good” via acts of kindness, good deeds, etc. (Keltner, 2009; Gray, 2011a,b); prosocial spending/donations (Aknin et al., 2013; Dai and Zhang, 2019)]. This paper provides a review of existing research and presents a coherent framework that illustrates ways technology can be used for the “good.” We then discuss how this framework can be used as a basis for future research in an understanding of positive usage of technology, the interplay between these factors, and psychological outcomes of these positive engagements with technology.

## Engaging with technology

Overall, technology use has expanded significantly over the past decade alone. Social media accounts for a significant portion of technology use. The numbers themselves provide some details about what is occurring. For example, we know *who* is using social media (e.g., 63% of users on TikTok in the United States are adults; Statista, 2022a). However, we do not know *how* these members are using technology and the granularity of what the effects of these engagements are on the individuals. Most of the recent research on technology has demonstrated several negative outcomes from extensive technology use, such as dependency, loneliness, issues involving privacy, social comparison, hate speech, anxiety, body dysmorphia, depression, and abuse (Thomée et al., 2010; Assimakopoulos et al., 2017; Laaksonen et al., 2020; Kakulla, 2021; Sutrisna et al., 2021; Danvers, 2022; Minadeo and Pope, 2022), but engaging with technology in the real world involves a complex system of simultaneous interactions that are less understood.

Within the United States, over 95% of young adults use social media (Auxier and Anderson, 2021). On average, Americans spend over 2 h using social media per day with over 81% of adults engaging in platforms such as YouTube (Suciu, 2021), which is more time than they spend sharing meals with others (Melore, 2021). Social networks such as TikTok have over 800 million global active users per month with over 37 million users belonging to Generation Z in the United States alone, and those numbers are estimated to increase to 48.8 million Gen Z users by 2025 (Statista, 2022b). Globally, more than 60% of young adults are able to access the Internet (Cerniglia et al., 2017; Sutrisna et al., 2021). Additionally, over 59% of Instagram users check the app daily and young adults spend, on average, over three hours per day on social networking platforms (Henderson, 2020).

The rapid rate of technology adoption and usage coupled with the advancement of technology has provided significant concerns for researchers, educators, and policymakers; these concerns span from what the nature of technology is to the fact that we lack sufficient understanding of how technology is used and how it impacts people’s lives in the short and long term (Pleasants et al., 2019; Krutka et al., 2022). Along with these concerns, more researchers have been focusing on understanding technology better, however, most have been focused on the negative consequences of technology use. This focus on negative outcomes may be because the strength of bad experiences is more powerful than the intensity of good ones (Baumeister et al., 2001). This occurs because the negative potency of bad experiences is much more salient than good events and heavily influences how individuals process bad experiences (Rozin and Royzman, 2001). This is especially true when individuals are using technology. The negative effects of technology are readily apparent when looking through research. For example, studies have shown negative outcomes of persuasive technology from algorithms that prioritize engagement at any cost (anger, anxiety, suicidal ideation, depression; body image negativity; Rhodes et al., 2020; 60 Minutes, 2021; Center for Humane Technology, 2021; Minadeo and Pope, 2022). The potency of negative engagement has devastating consequences on individuals across every age group (social comparison, distortions in self-perception, disconnection; addiction, social isolation/rejection, radicalization and distrust; Baumeister et al., 2001; Eisenberger et al., 2003; Kross et al., 2013; Fredrickson and Joiner, 2018; Costello et al., 2022; Wilson, 2022).

Previous research has demonstrated that when individuals endure negative experiences and emotions, the effects of these experiences in turn reflect a series of negative downstream consequences (e.g., anger, depression, fear, fight, or flight; Fredrickson and Joiner, 2002, 2018). Immediately, the effects of these negative emotions lead to a narrowing of action repertoires (anger, fear, hate, detachment) and an inability to connect with others. It also leads to internal manifestations of negative consequences such as anxiety, depression, withdrawal from others, susceptibility to disinformation and radicalization, and an inability to build long term resources that support the organism’s well-being (Fredrickson, 2003; Center for Humane Technology, 2021; Bor and Petersen, 2022; Regehr, 2022).

Algorithms used in social media, in an attempt to maintain the attention of end users, provide a constant barrage of sensations, images, videos, and other forms of outputs that effectively activate regions of the brain and influence cognitive attention and behaviors (Fogg, 2003; Godinho et al., 2017; Cohen, 2018). The regions that become highly activated and dysregulated are typically involved in responses such as addiction (ventral tegmentum), information processing (Prefrontal Cortex), or fight or flight response (limbic system) of the users who engage with these cues (Fogg, 2003; Seo et al., 2020; Center for Humane Technology, 2021). These negative experiences, elicited through highly adaptable artificial intelligence using machine learning, strategically target the neurocognitive systems and hijack the autonomic nervous system. Experts in technology refer to this advanced process as a “race to the bottom of the brainstem” [The Rubin Report (Director), 2017]. This engagement can lead to pervasive unintended consequences such as fear, anger, disgust, radicalization, hate, online bullying, alienation, and more. It is important to note that some studies have shown complex nuances, small effects, or mixed results in relation to social media use and well-being (Orben and Przybylski, 2019; Kross et al., 2020; McFarland et al., 2023). However, many investigations and reports have come to light which demonstrate how negative engagement with technology can lead to adverse consequences such as increased suicidal ideation, depression, anxiety, problematic social media use, violence, hate speech, and other consequences, including negative mental health effects in groups such as teenagers (Kavanagh et al., 2019; 60 Minutes, 2021; Castaño-Pulgarín et al., 2021; Huang, 2022; Shannon et al., 2022).

Other consequences of negative engagement with technology can include addiction, cyber bullying, and other misuses of devices that are harmful to people’s health such as extensive blue light exposure, sleep dysregulation, and aggressive behaviors from exposure to disinformation, angry provocative content and messages through networks, podcasts, and fear inducing viral videos (Neumann, 2013; Erreygers et al., 2019; Kırcaburun et al., 2019). These negative experiences are not just limited to social media. Human interactions with technology are leading to severe polarization and isolation in many individuals who are not adequately prepared to interact with technology in healthy ways. What if, however, there was an approach to supporting healthy engagement with technology and using it for the good? In order to answer this question, we need to first clarify what we mean by “the good” and engagement in *the good* through technology.

## Engaging with the good

Research on the *good* has expanded researchers’ understanding of how humans interact and shape their daily lives across developmental standards and expectations. The field of Psychology has yet to define the term *good*, but researchers studying positive psychology have demonstrated that good feelings are an essential component to well-being (Fredrickson, 2003; Seligman, 2011). For example, experiences that elicit positive affect broaden the scope of attention which in turn lead to the building of future resources which then provide numerous positive benefits that shape positive experiences and broadening repertoires (Fredrickson and Joiner, 2002, 2018; Lyubomirsky, 2010). Other researchers have demonstrated that good actions are also an essential component to well-being (Keltner, 2009). For example, directed acts of compassion or kindness, and active cooperation with others rewards a region of the brain known as the nucleus accumbens which is densely populated with dopamine receptors, and in turn enhances positive experiences.

Therefore, in order to understand what is meant when we use the term *good*, it is important to provide an operational definition of the word that is used to describe these positive terms. The etymology of the word good is derived from Germanic Origin gudą, and from old English gōd, implying virtuous or morally uplifting context. Oxford dictionary defines good as “useful, advantageous or beneficial in effect, possessing or displaying moral virtue, showing kindness, giving pleasure; (something that is) enjoyable or satisfying” (Oxford English Dictionary, 2022). Aristotle refers to the “supreme” good as an activity of the rational soul as it relates to virtue. Virtue, for the Greeks, is equivalent to excellence (Aristotle Bartlett and Collins, 2011). Within the beliefs of Mohism, Mohists advocate a consequentialist criterion for evaluating *good* actions (Mo and Fraser, 2020). What is benevolent or right is what provides *good* consequences—specifically, it benefits people. Among benefits, doing good for others, such as donating, volunteering, caring for, or feeding others takes priority over simple hedonic enjoyment. Mohists prize the virtue of benevolence, which they regard as committing us to furthering the benefit of all the world (including ourselves).

Venot and Veldwisch (2017) present a higher-level overview of what is *good*, stating, *“*Connections and associations are made to something that is Good in the abstract sense, or to values assumed to be universal (though they reflect a narrow vision of progress, mostly Western and male dominated), such as equity, progress, development, and modernity” (2017). These unique interpretations and presentations of what is *good* provide context around the term, however in order to understand what is *good* from a psychological perspective it must be understood within the context of how it is being used. Therefore, for the purposes of this paper we define *good* as a mechanism or association between positive interactions with authentic beneficial effects that contribute to positive outcomes. The relationship that humans share with technology is complicated and while there are some shared associations with experiences that elicit positive outcomes, there is an essential need to understand how humans are engaging in the *good* with technology. Based on previous research, engaging with the good can be categorized into three classifications: seeing the good, feeling good, and doing good.

### Seeing good

Seeing good is one way for people to engage in the good. Throughout the day, people are exposed to visual cues (events, actions and other behaviors, communication/information) that are meaningfully assessed through an intricate cognitive appraisal process. This is especially true when using technology. Operationally, seeing good involves a positive visual-cognitive top-down process where visual stimuli influence attention, personal expectations, and perceptual information (Gilbert and Li, 2013). When an individual perceives a positive visual cue, this, in turn, influences positive affective experiences and meaningful cognitive judgments (Diener, 1984; Eid and Larsen 2008; Hanson, 2013). Research demonstrates that the downstream consequences of seeing good leads to increased motivation and the development of strategies for secure social interactions, behaviors, and relationships (Sprafkin et al., 1975; Sanders et al., 2000; Janicke-Bowles et al., 2018; Gilbert and Basran, 2019). This is further evidenced through an evolutionary lens of human adaptation, where seeing the good relates to the capacity for positive experiences through cooperation and joy (Smith, 2010; Hanson, 2013; Gilbert and Basran, 2019). It has also been associated with other perceptions, such as experiences in novelty or perceptual vastness (awe; Rudd et al., 2012) and altruistic joy (the happiness from witnessing the good fortune of others; Hanson, 2013).

Some examples of seeing good include social or prosocial perceptions or seeing good in the lives of others (Smith, 2010). Other examples include inspirational visual cues (Haidt, 2000; Janicke-Bowles et al., 2018), imagining good facts (Hanson, 2013), having access to information that contributes to positive outcomes (Graham and Nikolova, 2012; Siegel and Thomson, 2017) and other visual experiences that increase accessibility/agency or positive emotions such as happiness, gratitude, awe, positive perceptions, or positive orientations (the general tendency to care about the needs of others; Thomson and Siegel, 2013; Thornton et al., 2019). Some researchers associate seeing good as a process of “taking in” visual and cognitive experiences as a means of coping and fostering well-being in their lives and the lives of others (Hanson, 2013). Essentially, seeing good is the access to visual information that contributes to positive outcomes. This visual process is associated with early evolutionary capacities for connections and interactions through positive non-verbal cues, such as smiling (Mukherjee et al., 2018).

Recently, research into visual stimuli within digital environments have reflected similar cognitive processes through digital visual cues. Positive cues from inspirational media can lead to the broadening of attention and perception (Haidt, 2003; Janicke-Bowles et al., 2018; Mukherjee et al., 2018), and prosocial outcomes (Greitemeyer, 2009). They are associated with increased motivation, positive emotions and inference of meaning (Gilbert and Li, 2013; Myrick et al., 2022). Positive visual cues during technology use include, but are not limited to, “good” things, such as funny videos, creative visual narratives, loving scenarios, prosocial video games, awe provoking content, fun experiences, and watching people do good things for each other (Sanders et al., 2000; Salimkhan et al., 2010; Thornton et al., 2019; Myrick et al., 2022). Essentially, seeing good is the access to information that contributes to positive outcomes.

### Feeling good

Feeling good is another way people engage in the good. Operationally, feeling good is associated with the presence of higher positive affective states. Positive affective states are defined as pleasant feelings that contribute to positive levels of hedonic well-being (more positive affect than negative affect; Diener and Diener, 1996; Diener and Seligman, 2002). Hedonic well-being is often defined as the process of seeking pleasure and maximizing good feelings (Waterman, 2008). A feeling is a subjective, evaluative process whose appraisal determines whether the feeling is pleasant or unpleasant (APA Dictionary of Psychology, 2022).

The appraisal process occurs through a biopsychological cascade of energy and interactivity across cognitive structures within the limbic system and prefrontal cortex (Rolls, 2005). The cognitive system integrates (subjective) information and in turn elicits good responses (Fredrickson, 2003). Feeling good increases hedonic levels and motivates humans to engage with their environments, build resources, connect emotionally, and engage with others in positive ways (i.e., approach behaviors; Diener and Diener, 1996; Belonging; Siegel, 2022). There are other mechanisms that contribute to experiences of good feelings. For example, a eudaimonic perspective (the actualization of one’s potential), emphasizes that positive feelings arise through the fulfillment and engagement in meaningful activities (Ryan and Deci, 2000).

Examples of activities that often elicit good feelings include but are not limited to, spending time with friends, relaxing, meditating (Diener and Seligman, 2002; Huebner et al., 2014), or engaging in stimulating activities (Holstein et al., 1990). Stimulating activities can include engagement with music, concerts and other events (through dancing, listening, singing, etc.; Dunbar et al., 2012), or playing games (Hunter et al., 2019; Gkogkidis and Dacre, 2020). With the convenience of technology today, people are able to relax or engage in stimulating activities from the comfort of anywhere and at any time around the world with devices. Other engaging activities with technology that contribute to good feelings, include viewing and receiving “likes” on social networking posts (Sherman et al., 2016; Ellis et al., 2020) and playing video games (Guegan et al., 2020). The benefits from positive or good feelings contribute to one’s quality and satisfaction with life and are correlated with an increase in people’s sense of “oneness” with others (Diener, 1984, 1994; Edinger-Schons, 2020; West et al., 2021), the building of trust with acquaintances (Dunn and Schweitzer, 2005), increased resilience (Fredrickson, 2003), and other mechanisms that increase positive resources. Given that technology has become a vessel for young adults to experience biopsychosocial cascades of good feelings through digital experiences (i.e., social media, video games, blogs, podcasts, etc.; Magis-Weinberg et al., 2021; Myrick et al., 2022), provide a pivotal role in providing additional opportunities for positive outcomes (Kushlev et al., 2021). The downstream effects from feeling good during positive technology use provide increased perception of peer support (Magis-Weinberg et al., 2021), inspiration (Meier and Schafer, 2018), motivation (Janicke-Bowles et al., 2018), prosocial behavior (Kushlev et al., 2021) and can even improve how people visually perceive the world and how they behave offline (Jolij and Meurs, 2011). Feeling good during technology use, therefore, represents another domain within the trichotomy of engaging in the good with technology, and may play a significant role in positive technology use.

### Doing good

Doing good is viewed as any action seeking to promote perceived positive outcomes. Actions involving directed compassion and kindness, or affiliative behaviors, are most often associated with the construct of doing good (George and Brief, 1992; Tappin and Capraro, 2018). Affiliative behavior is an important component of doing good and is defined as a positively interpreted action that facilitates peaceful and friendly interactions (Depue and Morrone-Strupinsky, 2005). Doing good is often accompanied by the concept of doing good deeds (Gray, 2011a,b). Good deeds are correlated with proactive agency, that is, a sense that an individual is motivated to construct, contribute, or influence circumstances through their choices and actions (Ryan and Deci, 2000; Bandura, 2006; Gray, 2011a,b; Pettengill, 2020). Within this study, “doing good” is viewed as any action seeking to promote positive outcomes. There is a reciprocal relationship between doing good and positive experiences, thoughts and behaviors, particularly when humans are able to cognitively assess their perceived impact. That perceived impact is a judgment that one’s actions have consequences for the welfare of others (Grant, 2007). As people do good, the impact of their actions influences their thought patterns and perceptions (Bower, 1975; Neisser, 1976; Bandura, 1989; George, 1991; Gray, 2011a,b), which reinforces the actualization of good behaviors.

Examples of doing good include but are not limited to, acts of kindness, donating, volunteering, promoting and posting positive content or comments on digital platforms (Pettengill, 2020), sharing authentic information or experiences, preparing for timely responses to crises and supporting people in need (Palen et al., 2007; Schueller et al., 2019; Hunsaker et al., 2020; Schueller and Torous, 2021; Tygielski et al., 2021), or sharing positive computer-mediated communication (Riva, 2002; Al-Zoubi and Shamma, 2021; Cavalheiro et al., 2022; Walsh et al., 2022).

Doing good leads to further perceptions of one’s agency and the impact of those actions, because perceptions of actions are the means by which people make sense of experience (Smith and Ellsworth, 1985; Gray, 2011a,b; Pettengill, 2020). Through the use of technology, people are able to participate more easily in topics they are passionate about and, in some cases acting as agentic influencers, by sharing and having access to authentic information and knowledge (Goldman et al., 2008; Dahal et al., 2020). These factors increase perceptions of agency and increase quality of life as opportunities to express feedback about social interests and other areas of concern positively influence motivation and other perceptions such as satisfaction with life (George, 1991; Ryan and Deci, 2000, 2001; Wessels, 2013).

Other benefits of doing good include individual and larger social advantages, including increases in positive affect, optimism, gratitude, life satisfaction, and joviality (Alden and Trew, 2013; Pressman et al., 2015). When accounting for the recipients of good deeds, research demonstrates that there are increases in positive mood and nonverbal cues such as smiling, which enhances the supporting nature of connections with groups (Gray, 2011a,b; Pressman et al., 2015). Doing good also influences the perceived impact of good behaviors and facilitates perceptions of self-efficacy, which in turn influences human agency and further actions (Depue and Morrone-Strupinsky, 2005; Bandura, 2006; Grant, 2007; Hawkley et al., 2007). Doing good for others fosters positive perceptions by others, which also contributes to feelings of agency, and positively influences human capabilities (Gray, 2011a,b). Additionally, good deeds create secure social interactions and supportive relationships, in addition to providing experiences of personal fulfillment (Ryan and Deci, 2001; Gray, 2011a,b; Siegel, 2022). Studies on individuals suffering from high social anxiety have demonstrated that doing good consistently over time also decreases social anxiety, increases relationship satisfaction, and significantly boosts positive affect (Alden and Trew, 2013). All of these benefits increase opportunities for positive experiences for “doers” and “receivers” demonstrating several positive outcomes.

## Engaging in the good with technology: a conceptual framework

**Figure 1:**
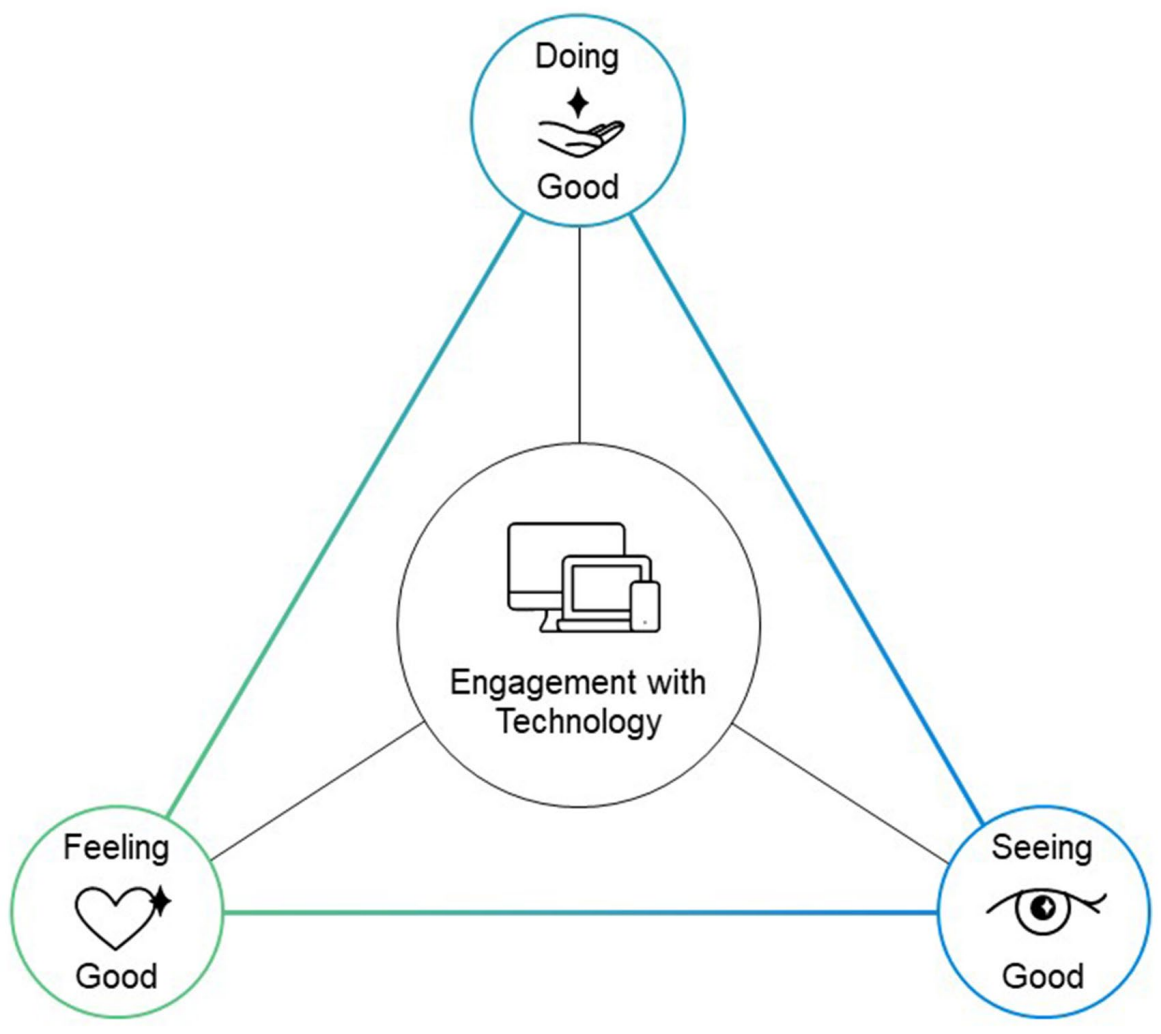
Engagement in the Good with Technology (EGT) Framework. The EGT framework reflected in this figure demonstrates three domains for engaging in the good. At each corner is a node that is influenced by positive experiences that may occur during technology use. Each domain is activated through engagement with technology as represented in the center of the figure. Depending on the type of engagement, each node can become highly activated or less activated depending on what is occurring. These changes are dynamic. Increases in one node may influence the others, inversely, decreases in one may show decreases in others and they may be bidirectional.

Modern Technology provides opportunities for individuals to engage in the *good*. Based on the three directions of research taken on the investigation of engagement in the *good* (seeing good, feeling good, doing good) we propose a novel conceptual framework that situates these domains of engagement in the good within the context of technology use. Figure 1 reflects our Engagement in the Good with Technology (EGT) framework as a triadic model. Placed at each corner of this triangle is one of the three domains of engagement with good. We discuss these three domains in the context of technology use as reflected centrally in the model. As seen in Figure 1, all domains of engagement in the good with technology are connected with lines, depicting the interrelated nature of these domains. The underlying premise of this model is that the elements of engagement in the good with technology are dynamic–they change across time and at times co-occur–and create a system. Namely, we deem this model as a dynamic network in which all elements of the network are interrelated and change as a system: Changes in one can be highly related to changes in other elements in the network of EGT and these relationships are proposed to be bidirectional.

In graph theory (Barnes and Harary, 1983) networks demonstrate the connectivity among “actors” that can be objects, people, items or any other groups of elements that form a system (Wasserman and Faust, 1994). Networks are made of elements that are denoted as “nodes” connected via lines, dubbed as “edges.” Edges represent the relationships among the nodes and illustrate the strength of the relationships among the nodes; these relationships can be directional or non-directional. Through a network perspective, we can examine relationships among all nodes of a network at the same time and explore changes in the configuration of a network over time and as a result of perturbations. Network analytics also provides the possibility of assessing the importance or “centrality” of each node in a network. For example, we can identify which node in the network is more strongly connected to all other nodes in the network (i.e., strength centrality) or acts as a connector among all the nodes of the network (i.e., betweenness centrality).

**Figure 2:**
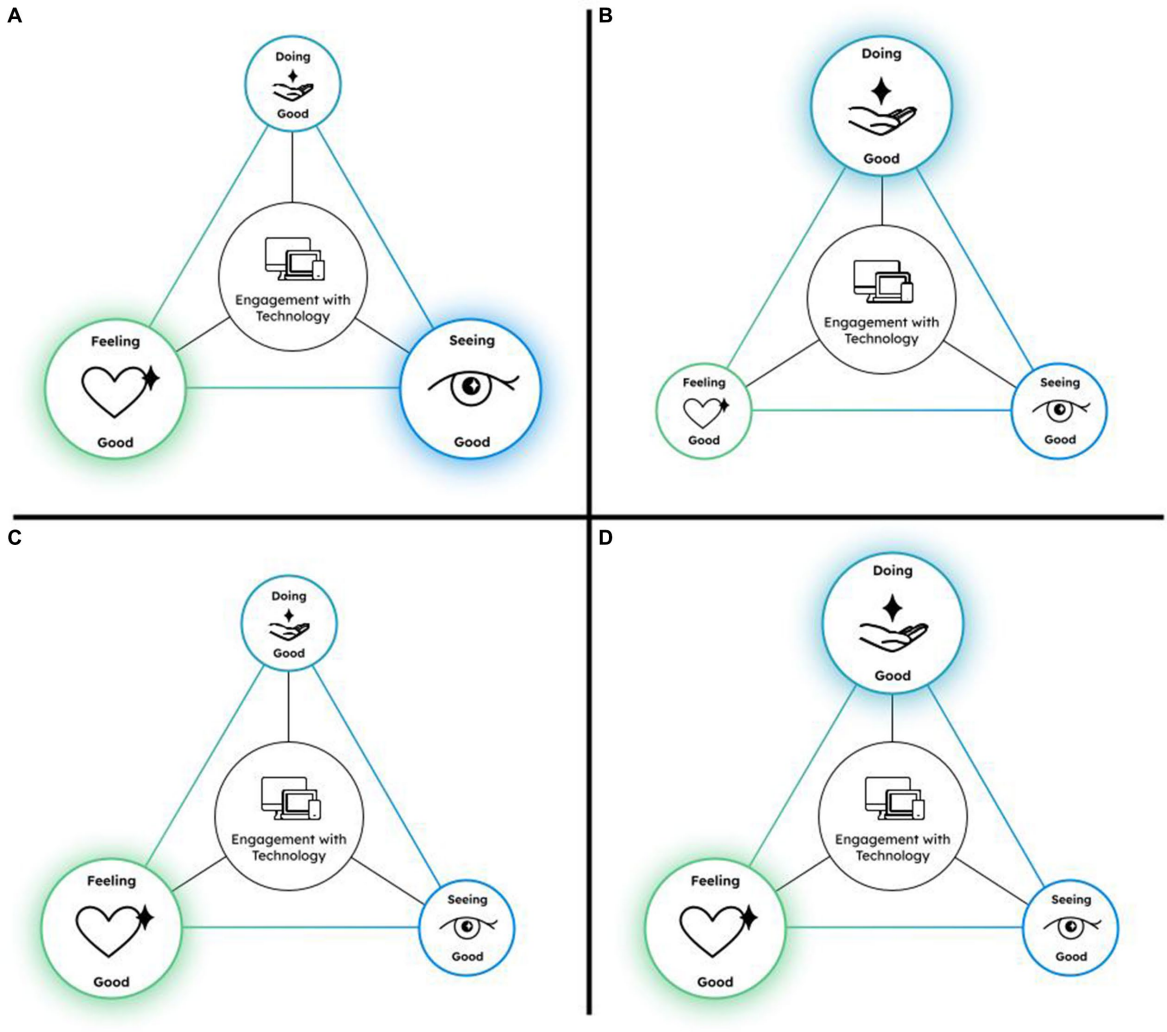
Example configurations of the EGT network model. Four different possibilities in network configurations are presented in this figure. Model **A** shows when Seeing Good and Feeling Good are increasing but Doing Good is not activated. Model **B** reflects when Doing Good is increasing and Seeing Good and Feeling Good are not activated. Model **C** demonstrates when Feeling Good is increasing but Seeing Good and Doing Good are not activated, Model **D** illustrates when Doing Good and Feeling Good are increasing, but Seeing Good is not activated.

Approaching the EGT model as a network, the three facets of engagement in the good with technology are considered as nodes and the lines connecting them are the edges quantifying the relationship among them. Through this, we can explore the interconnectivity as well as the importance of the nodes within the network. Moreover, we can explore the EGT model in terms of the different configurations the network can take and how changes in one node of the network in different contexts can change the configurations of the network in different ways. Figure 2 demonstrates examples of the different configurations the EGT network can take.

Figure 2A demonstrates an example configuration of the EGT network where, for example, someone is seeing good by watching an act of kindness video on their smartphone. While seeing good is increasing in this triadic network, feeling good is also increasing because the video is making them feel happy. Thus, in this model, the two seeing and feeling good nodes are activated (depicted via their increase in size) but doing good is less relevant and therefore, smaller in size. While the act of seeing good has the potential to increase people’s motivation to do good (Janicke-Bowles et al., 2018), in this specific example, doing good has not yet been activated.

Figure 2B on the other hand, demonstrates an example of the EGT network configuration where someone engages in doing good, for example by donating funds to charity for war refugees on their computer (increase in doing good). While doing that, the individual may encounter war images of violence occurring in the war, leading to negative feelings (decrease in feeling good). Seeing good in this case is also small and less activated due to the imagery that they are witnessing in this context.

Figure 2C depicts a third possible configuration of the EGT network. In this case, an individual could be listening to music and their favorite song comes up. They feel good and begin dancing. In this case, the individual’s feeling good is heightened while their levels of Seeing good and doing good may remain the same.

Figure 2D represents another configuration where doing good and feeling good is elevated but seeing good is inactive. An example of this can be when someone uses social media to message a friend who is going through a tough time. While supporting their friend, the person feels good about being a source of support. In this case, this person has low exposure to seeing something good using their technology.

Network configurations for the EGT model are not limited to the ones presented in Figure 2, rather they represent examples of ways that this model can be adopted to quantify various scenarios of engagement with the good through technology in terms of the three domains in different life contexts. Quantifications of these configurations through network analysis can then be examined in relation to psychological outcomes of interest, further elaborated in the next section.

### Applications of the EGT framework

There has been a request from researchers, educators, and policymakers to help people flourish with technology (Kushlev and Leitao, 2020; 60 Minutes, 2021; Kamenetz, 2021; Minadeo and Pope, 2022). Yet, there needs to be more research on how people engage in the good with technology. Beyond the theoretical and evidentiary support, the EGT framework provides a conceptual and theoretical grounding across three domains that can be used to explore positive technology experiences. Specifically, this model provides a dynamic and systems-level structure for understanding engagement in the good with technology. We take a network perspective toward this model where the different domains of engagement with the good via technology are assumed to be interconnected and should be examined as a whole instead of the sum of its parts.

Using the EGT Framework, future research can examine the dynamical changes in the EGT network in relation to various psychological and health outcomes. In fact, through the network perspective, we can bypass examining the individual impact of each of the domains of good technology use with mental health outcomes but rather explore the different configurations of the EGT network as a whole with outcomes of interest (see, e.g., Heshmati et al., 2021). For example, frequent use of technology to see good throughout the day (seeing good) may also stimulate good feelings in the person (feeling good) and ultimately lead to altruistic inclinations and prosocial motivation (doing good). This makes a “closed” triadic network where all three edges of the network are present (Robins, 2015); this is as opposed to an open triadic network that has at least one edge missing from the network (i.e., no connectivity or association between two nodes of the network). With this, we can examine whether a closed triadic EGT network (all three aspects of engaging in the good are adopted simultaneously and increased together) is predictive of a person’s satisfaction with life as opposed to an open triadic network (only one or two aspects of engaging with the good is being adopted).

Moreover, taking a network approach toward domains of positive technology use can be helpful in informing future interventions targeted at increasing technology use for the good. Through measures of network centrality (e.g., strength, betweenness, closeness) we can quantify the importance of each of the three different domains of good in the EGT network. In other words, we can identify which node (domain of the good) in the network is most strongly connected to the rest of the network—namely, increases in that node will make it highly likely that other nodes in the network would increase as well. This particular node can then be the point of influence in this network for interventions since it is the most central and highly connected to other nodes. This would make the intervention more economical such that with increases in one part of the network, other aspects are also likely to increase.

### EGT as a research tool

This Model can serve as a resource in both research and design landscapes. From a research standpoint, this model can be used to empirically test theoretical assertions relevant to positive technology use. For example, in emotion research, a ratio of positive events to negative events has been proposed as a means of overcoming the effects of negative experiences (Fredrickson, 2009; Lyubomirsky, 2010; Rusu and Colomeischi, 2020). Even though a debate exists around the value of the ratio (Gottman and Gottman, 2015; Friedman and Brown, 2018), research continues to support the notion that it is important for humans to experience more positive experiences than negative experiences in order to flourish (Lyubomirsky, 2010; Rusu and Colomeischi, 2020). As a Research tool, people can use the EGT framework to examine the amount of positive engagement with technology in relation to negative engagement. This model could serve as a means of understanding the degree to which people are engaging in positive experiences and weighing them against negative ones. This tool could be constructive in advancing the development of measures that assess positive technology use. By providing tangible representations of technological interactions, they also serve as cornerstones for future studies, enabling a deeper exploration into the essence of technology use.

Moreover, investigative inquiries using this framework could be tailored to discern the antecedents of positive technology use, thereby shedding light on key variables that influence user interactions and outcomes. This model could also be used to develop interventions that support positive outcomes by dialing up the degree or frequencies of positive engagements and then measuring how these may impact individual responses to them. This framework can also be used to research technology users across generational cohorts. For example, we know technology use is pervasive in young adults (18–35). Therefore, it could be beneficial to know how much they use technology for good, what modes they use, and how positive technology use is related to their well-being.

Other areas that could be explored include whether these cohorts use technology for the *good* through seeing, feeling, and doing good. If so, how often are they engaging in seeing, feeling, and doing good in their daily lives? What modes are they using to engage in these behaviors (e.g., messaging, virtual reality, gaming, social media, sharing)? Other questions that could be explored include whether people who engage in the good with technology frequently report higher subjective well-being. Or is engaging in the good through seeing, feeling, and doing via technology related to higher levels of trait altruism and prosocial behavior? These are just some of the questions that would be interesting to explore in further research using the EGT framework.

### EGT as a design tool

Many researchers have urged technology designers (Technologists) to take decisive action. For example, some researchers are requesting strategies for improving positive engagement (e.g., body positivity; authentic information) with technology among populations who find it challenging to deal with exposure to sensitive content on social media platforms (Minadeo and Pope, 2022). Integrating these components into applications spanning diverse platforms and virtual experiences may yield advantageous results for end users of these platforms. Considering that one of the primary goals of technology companies is to enhance user engagement and increase corporate investments, the EGT Model offers a unique avenue for technologists. It allows them to establish protective mechanisms or procedures with algorithms that might positively affect users by boosting positive emotions, resonance, and engagement.

Take, for instance, a situation where a social media algorithm persistently recommends potentially harmful content. With the EGT Framework, it could establish safeguards where, through machine learning, the algorithm could start proposing content that typically promotes beneficial or affiliative behaviors. This might encompass exposure to uplifting videos, options to contribute to virtuous causes, and subsequently reflecting those advantageous outcomes to the user. Consider a donation scenario that also provides insights into the favorable repercussions of such an act. Should individuals integrate a direct beneficial outcome into their cognitive processes, they are enabled to not only Do Good and See Good but also to Feel Good.

Another potential function of the EGT Network lies in its capacity to foster positive engagement through the promotion of enriching learning environments. Given the overwhelming volume of information readily available today, it can be challenging for individuals to discern and comprehend genuinely helpful and authentic information. The EGT Network can counter this issue by creating positive spaces or illuminating pertinent and healthy information. This approach promotes beneficial outcomes through productive communication, such as reframing and reliance on fact-based sources. Additionally, by supporting healthy behaviors, the EGT Network could enhance users’ learning abilities. For instance, when end-users seek information about healthy exercise or diet suggestions, resources designed with an EGT Framework can be particularly beneficial. Such resources can guide users to authentic information from professionals, connect them with positive and healthy support groups or mentorship opportunities, and even allow them to support others on similar journeys. Consequently, this enables users to make well-informed decisions.

Given that the frequency of interactions and the amount of time that people engage with devices is increasing exponentially, future research could benefit from a framework that reflects the dynamics of positivity-focused technology strategies across different technological landscapes such as Extended Reality (XR; Virtual Reality, Augmented Reality), gaming, metaverse environments, or more. Understanding how the EGT Network can function across interactions is an important element in supporting future applications. As evidenced, incorporating elements across different platforms could promote positive behaviors such as cooperation and mutual support or create positive learning environments for all ages.

## Conclusion

Technology, as always has been the case, is only going to become further integrated into the human experience. Research has demonstrated that at times, how we use technology can reduce perceptions of agency, narrow our perceptual scope of attention, and disconnect us from one another. As we have demonstrated in this article, how we choose to use technology is ultimately the main predictor of how it impacts us. However, currently, we lack the tools required to measure and understand how we may consciously choose to engage with our technology, positively. Therefore, measuring and having instruments at our disposal that supports adaptation to technology can empower how people engage with technology in positive ways and promote human flourishing.

By considering the broad scope of how good is enacted with technology we can provide more information about the positive influences and directions of positive technology use. In view of the fact that negative technology experiences are a common occurrence for everyone, having a coherent reference point for how positive engagement occurs, may bolster support for those who need it most. Whether people are text messaging, exploring metaverses, using apps, video conferencing, using social media, or more, technology plays a vital role as an extension of the human experience. The triadic model for engaging in the good with technology (i.e., EGT framework) provides a coherent framework and important context for interactions and encourages further exploration of positive experiences with technology use. This framework further supports the exploration of research questions that have not been answered before. Examining positive interactions of technology provides an opportunity for researchers, educators, and practitioners to understand how to support and enhance well-being in populations, and determine successful methods for people to engage with technology in positive ways. This model can be used and adopted by researchers, organizations, companies, institutions as a means of understanding how to enhance positive upward spirals in people’s mental health through *good* technology use.

## Author contributions

AV was an overall majority of this paper, developed the theory, conceptual framework, researched, wrote the literature review of research, and proposals. SH was a valuable resource involved in discussing the framework, reviewing the work, editing and reviewing the paper, contributed expertise in her knowledge of Social Network Analysis to help inform the supporting analyses, and proposed applications. All authors contributed to the article and approved the submitted version.

## Conflict of interest

The authors declare that the research was conducted in the absence of any commercial or financial relationships that could be construed as a potential conflict of interest.

## Publisher’s note

All claims expressed in this article are solely those of the authors and do not necessarily represent those of their affiliated organizations, or those of the publisher, the editors and the reviewers. Any product that may be evaluated in this article, or claim that may be made by its manufacturer, is not guaranteed or endorsed by the publisher.
